# Friends because of foes: synchronous movement within predator–prey domains

**DOI:** 10.1098/rstb.2023.0374

**Published:** 2024-09-04

**Authors:** Christina M. Prokopenko, E. Hance Ellington, Alec Robitaille, Jaclyn A. Aubin, Juliana Balluffi-Fry, Michel Laforge, Quinn M. R. Webber, Sana Zabihi-Seissan, Eric Vander Wal

**Affiliations:** ^1^ Department of Biology, Memorial University of Newfoundland, 232 Elizabeth Ave, St. John’s, NL A1B 3X9, Canada; ^2^ Range Cattle Research and Education Center, University of Florida, 3401 Experiment Station Rd, Ona, FL, USA; ^3^ Cognitive and Behavioural Ecology Interdisciplinary Program, Memorial University of Newfoundland, St. John’s, NL, Canada

**Keywords:** predator–prey habitat domains, movement synchrony, elk (*Cervus canadensis)*, caribou (*Rangifer tarandus*), multi-predator system, functional traits

## Abstract

For prey, movement synchrony represents a potent antipredator strategy. Prey, however, must balance the costs and benefits of using conspecifics to mediate risk. Thus, the emergent patterns of risk-driven sociality depend on variation in space and in the predators and prey themselves. We applied the concept of predator–prey habitat domain, the space in which animals acquire food resources, to test the conditions under which individuals synchronize their movements relative to predator and prey habitat domains. We tested the response of movement synchrony of prey to predator–prey domains in two populations of ungulates that vary in their gregariousness and predator community: (i) elk, which are preyed on by wolves; and (ii) caribou, which are preyed on by coyotes and black bears. Prey in both communities responded to cursorial predators by increasing synchrony during seasons of greater predation pressure. Elk moved more synchronously in the wolf habitat domain during winter and caribou moved more synchronously in the coyote habitat domains during spring. In the winter, caribou increased movement synchrony when coyote and caribou domains overlapped. By integrating habitat domains with movement ecology, we provide a compelling argument for social behaviours and collective movement as an antipredator response.

This article is part of the theme issue ‘The spatial–social interface: A theoretical and empirical integration’.

## 1. Introduction

Predator–prey interactions are an ecological linchpin of system dynamics. Co-evolution with predators has spurred prey to develop a variety of strategies to mediate predation risk, consequently having broad ecosystem impacts [1]. For example, spatial and temporal avoidance of predators by prey through changes in movement and habitat association influences the areas used by both predators and prey [2,3]. Furthermore, movement decisions by individual prey are intertwined with higher-level social behaviours or collective patterns such as aggregation [4] that themselves can be strategies for reducing predation risk [5]. When considering spatial patterns of predator–prey dynamics, it is useful to invoke the concept of a habitat domain—defined by [6] as ‘the spatial extent of habitat space that predator and prey use during their foraging behavior’. Furthermore, the domain of a predator is interrelated to the hunting mode of a predator. Predator hunting modes describe strategies used to search for and capture prey [7], e.g. ambush predators use stealth and surprise to quickly capture prey whereas cursorial predators actively chase prey. Typically, we expect active cursorial predators to have a broader domain and for prey to respond accordingly [8]. Here, we integrate community ecology concepts, such as habitat domains and predator hunting modes, with spatial–social analyses to test the contexts in which collective movement of prey is an antipredator response in contrasting ungulate–predator systems.

Social behaviour can be an antipredator strategy [5], where prey reduce predation risk through group vigilance [9], dilution [10] or even confusion [11]. Social animals in the same group move together; within these groups, we define movement synchrony as the conformity in direction and speed between two animals. Collective group movement builds from dyad-level movement synchrony [4]. Movement synchrony at the dyad level and collective movement at the group level can facilitate information transfer about resources, competition and risk of predation among group members [12]. Furthermore, movement synchrony maintains a group through space and can render risky places less so through increased detection through shared vigilance [13,14]. Predation is therefore a major driver of movement synchrony at the dyad level or collective movement at the group level [13,15]. Collective movement is an antipredator behaviour that could alleviate predation risk and prey vulnerability.

Spatial patterns of predator–prey dynamics are an active space race. Predators are motivated to use areas on the landscape where prey availability and catchability are maximized [16,17]. Predators may select landscapes perceived as linked to prey’s food resources in an attempt to increase prey availability and catchability [18,19]. For prey, spatial overlap with predators puts the avoidance of risky places at odds with acquiring resources, causing prey to compromise immediate energy intake with survival or to enact additional tactics to reduce their predation risk while continuing to share space with predators. When prey forage in the same spaces that their predators hunt, habitat domains overlap which spatially conflates risk and rewards among both the predator and prey (i.e. joint-spatial anchor [3]). For example, the joint-spatial anchor in predator–prey domains benefitted puma hunting their vicuna prey and vicuna shifted spatial behaviour as an antipredator strategy [3]. Descriptions of antipredator responses can benefit from incorporating the broader spatial and environmental context of predator and prey habitat domains.

Prey vulnerability is also an important factor when describing their antipredator responses. Prey can assess and counteract their own vulnerability to predators through many tactics including detection, evasion and injury [20]. For example, more vulnerable penguin chicks fled sooner and farther distances when approached [21]. Variation in antipredator responses can be driven by vulnerability in offspring as illustrated by adult mule and white-tailed deer exhibiting different antipredator responses when with vulnerable young [22]. Prey antipredator behavioural responses can depend on both intrinsic qualities of the prey and the predator, for example, predator hunting mode [23]. Grasshoppers, for example, contracted their domain size and moved less in response to sit-and-wait spiders but moved more and did not contract their domain size in response to spiders with active hunting modes [8]. Similarly, ungulate prey varied their behavioural response with predator hunting mode. Ungulates were more vigilant when exposed to cursorial predators but not ambush predators and also decreased time in risky areas with ambush predator cues but not cursorial predators [24]. Therefore, within areas where the predator and prey habitat domains overlap, the emergent socio-spatial responses are expected to vary with the vulnerability of prey and hunting modes of predators.

We conducted a scoping literature review to summarize the current understanding of prey sociality and space use strategies in response to predation risk and found evidence that gregarious prey use conspecifics to mediate predation risk (electronic supplementary material, appendix 1). From this review, we hypothesized that synchronous movement within groups reduced the risk of predation. Synchronous movement as an antipredator response could be sensitive to spatial variation in predator and prey habitat domains, traits of the prey (age-dependent vulnerability) and predator type (guilds, hunting modes). We examined how predator and prey habitat domains affect movement synchrony of prey in two systems: Newfoundland, Canada, which includes caribou (*Rangifer tarandus*), coyotes (*Canis latrans*) and black bears (*Ursus americanus*); and Manitoba, Canada, which includes elk (*Cervus canadensis*) and wolves (*Canis lupus*). The predators in these systems vary in hunting modes. Wolves and coyotes are both coursing predators but pose different levels of risk to large ungulate prey species [25] in this study. In general, wolves target larger ungulate prey in their diet than coyotes [25]. While bears are predators in this system targeting young calves during the spring, classified here as ambush as their chases occur over short distances and periods [26,27]. Indeed, there is seasonal variation in the vulnerability of ungulate prey to predation. Specifically, the ability to resist acute predation through flight or defense can depend on prey age, e.g. ungulate neonates have reduced mobility [28].

We tested for changes in prey movement synchrony in response to prey foraging domain and predator hunting domain. We expected, regardless of predator hunting domain, prey would move more synchronously in foraging domains. Within the predator hunting domain, regardless of prey foraging domain, we expected prey to move synchronously as a social antipredator strategy. Finally, we expected the pressures to eat and not be eaten would be greatest in areas where both predator hunting domain and prey foraging domain overlap. We predicted that prey would move most synchronously in these areas of predator–prey domain overlap to maintain their groups and reduce risk. We predicted that prey movement synchrony would be more common in habitat domains of active coursing predators relative to ambush predators. The reason for this is twofold: movement synchrony in response to active predators facilitates sharing risk detection through group vigilance and dilutes risk when evading an encountered predator. Our work intends to be a compelling test of prey social responses to predators through integrating prey vulnerability and predation hunting modes with spatial habitat domains.

## 2. Methods

### (a) Study areas

#### (i) Riding Mountain National Park, Manitoba

We studied elk and wolves in Riding Mountain National Park (RMNP) in southwestern Manitoba, Canada from 2008 to 2018. Southwestern Manitoba has a continental climate with large annual variations in temperature and considerable precipitation. RMNP consists of eastern deciduous, boreal, mixed-wood forest, grasslands and marshlands. The dominant forest cover species are aspen (*Populus tremuloides*) and conifer (*Picea glauca, Picea mariana* and *Pinus banskiana*), often with dense understory (*Corylus cornuta* and *Crataegus chryscocarpa*). RMNP contains many open water bodies including creeks, ponds and lakes.

During the study period, the elk population in RMNP was under active management to reduce the transmission of bovine tuberculosis ([29; *Mycobacterium bovis*) leading to a population decline (2100 to 1200 animals from 2008 to 2017; Parks Canada, unpublished data). In the RMNP ecosystem, wolves are the main predator of elk. Wolf populations have remained relatively stable during the study period, with an average population of 77 animals within the park (with a peak of 113 animals in 2011; Parks Canada, unpublished data).

#### (ii) Middle ridge wildlife reserve, Newfoundland

We studied adult female caribou, coyotes and black bears in the Middle Ridge Wildlife Reserve (MRWR) on the island of Newfoundland, Canada between 2008 and 2014. The Island of Newfoundland has a humid-continental climate and persistent precipitation throughout the year. The MRWR is composed of coniferous and mixed forest dominated by balsam fir (*Abies balsamea*), black spruce (*P. mariana*) and white birch (*Betula papyrifera*) as well as bogs with stunted black spruce and tamarack (*Larix laricina*). Barren rocks, lakes and ponds are also common land features.

Caribou in Newfoundland have undergone both increases and decreases in abundance over the last 60 years [30]. The estimated abundance of the Middle Ridge caribou herd between 2008 and 2013 ranged from 8782 to 10 445 (Newfoundland and Labrador Department of Environment and Conservation, unpublished data). Coyotes and black bears are the primary predators of caribou calves in Newfoundland, while coyotes are the primary predator of juvenile and adult caribou in winter [31,32]. Black bears are native to Newfoundland, while coyotes were first recorded in western Newfoundland in the 1980s [33].

### (c) Habitat domains

Habitat domains are often described as discrete areas measured from direct observations. Previous studies on predator and prey habitat domains of collared large mammals have used habitat selection analysis, often with a resource selection function approach, to create a continuous definition of habitat domain across the landscape. Furthermore, predator hunting domain has been articulated using known diel periods, predator activity or kills [3,34]. Similar to the data in these studies, our focal species used large spatial areas and were imperfectly monitored, such that areas used were only known when GPS fixes were collected.

In accordance with the original concept of habitat domains and previous work, we sought to identify prey and predator habitat domains as a continuous value across the landscape, such that any given area on the landscape had an estimated species-specific habitat domain value. Moreover, we acknowledged that not every location where an individual was known to occur (through GPS fixes) would be representative of that individual’s habitat domain, so we sought to delineate GPS fixes into specific movement states that are hypothesized to be associated with resource-acquisition periods of prey and predators (foraging and hunting, respectively). To estimate the relative probability that any given area of the landscape was the prey or predator habitat domain, we: (i) collected GPS telemetry location data; (ii) estimated species-specific movement states as an index of behaviour; (iii) conducted season and behaviour-specific resource selection functions (bRSF); and (iv) predicted season- and species-specific continuous habitat domain values across the landscape using bRSF models.

### (d) GPS telemetry data

In the RMNP study area, we used GPS location data of 38 adult female elk from 2008 to 2016 and 23 adult wolves from 2016 to 2018 that were captured as part of an ongoing research and monitoring program. Wolf and elk GPS collars (Lotek, Televilt, Sirtrack and Telonics) were programmed to record locations every 2 hours or were rarified to this rate. Animal captures in RMNP followed Memorial University of Newfoundland Animal Care Protocol #16-02 EV.

In the MRWR study area, we used location data of 41 adult female caribou from 2009 to 2013, 10 adult coyotes from 2008 to 2014 and 42 adult black bears from 2008 to 2013 that were captured as part of a larger research and monitoring program. Caribou, black bear and coyote GPS collars (ATS, Lotek and Telemetry Solutions) were programmed to record locations every 1, 2, 4 or 8 hours, depending on species, year and season. Animal capture and handling procedures in MRWR conformed to guidelines established by the American Society of Mammalogists [35].

We projected fixes to Universal Transverse Mercator (UTM 21N for Newfoundland and UTM 14N for Manitoba) and calculated the step length and movement rate for each individual using R [36]. We removed potentially erroneous fixes based on a movement rate filter (10 km/h for black bear and 20 km/h for other species).

### (e) Estimate species-specific movement states

To classify GPS fixes into movement states we used the Hidden Markov models (HMM [37]). We fit our GPS fixes into regular trajectories by rounding our location data to the nearest 15 min interval. We then split these regular trajectories into individual bursts when there were two or more consecutive missed fixes. We applied a continuous-time correlated random walk model to predict missing locations within each burst (equivalent to single imputation [38]). We then used the bivariate time series of step lengths and turning angles from these trajectories to generate movement models based on two movement behaviour states with HMM using the R package momentuHMM [39]. We modelled step lengths using a gamma distribution and modelled turning angles using a wrapped Cauchy distribution. We generated predictive movement behaviour states for each step (location) using the best models for each number of movement behaviour states using the Viterbi algorithm to decode the underlying unobserved Markov chain [37]. We then removed any imputed steps (from the continuous‐time correlated random walk model). These methods are somewhat robust to missing data, however, we have found that performance declines with increasing proportions of missing data within bursts or with bursts with fewer fixes, thus we excluded bursts with ≥20% missing fixes and required bursts to cover a period of at least 10 days.

We assumed the two-state movement model would delineate two movement behaviours: encamped and moving. Encamped behaviour was characterized by short step lengths and high turning angles. For predators, encamped behaviour was probably related to resting, whereas for prey was probably related to foraging (and thus our target behaviour for the prey foraging domain), although some prey behaviours not related to active foraging such ruminating would also be related to encamped behaviour. Moving behaviour was characterized by long step lengths and low turning angles. For prey, moving behaviour was probably related to travelling between foraging sites, whereas for predators was probably related to hunting (and thus our target behaviour for the predator hunting domain), although some predator behaviours not related to active hunting such as travelling between resource patches or patrolling territories would also be associated with moving behaviour. Ultimately, given the coarseness of our GPS data, we cannot fully isolate foraging and hunting behaviours from all other behaviours for prey and predators, respectively. We can, however, isolate these broader movement states and thereby reduce some of the noise that would have been present otherwise.

### (f) Behaviour-specific resource selection functions

With our prey foraging behaviour and predator hunting behaviour identified, we next generated bRSF models using logistic regression and the glm function in program R for each species in two distinct seasons: winter (1 January–13 March) and spring (21 May–31 July). Our seasonal delineations were based on snow conditions, seasonal prey movements and predator behaviour (winter) and calving period of the prey species (spring). Note, black bears den during the winter in Newfoundland, so we only generated a spring black bear bRSF.

For our bRSFs, we defined the extent of available areas by applying a 200 m buffer around the 100% minimum convex polygon of all GPS fixes within each season of each species where we identified either foraging (prey) or hunting (predator) behaviour. Note, for RMNP the available areas for both wolf and elk were clipped to the park border because there is an abrupt change from undisturbed natural habitat to agriculture and human development. We identified available locations within our area of availability by regularly sampling locations at 200 m intervals [40]; we report the number of used and available locations for each species and season bRSF model in electronic supplementary material, appendix 2.

To keep the selection values of predator and prey habitat domains comparable within a study area, we ran one global bRSF model for each season and species, although the land cover covariates in the global model differed between the two study areas. The land cover covariates in the global model for the RMNP study area included bog, conifer forest, marsh, mixed-wood forest and open deciduous forest (30 m resolution; Manitoba Remote Sensing Centre 2004). The land cover covariates in the global model for the MRWR study area included conifer scrub, forest, lichen and rocky or barren ground (30 m resolution; NLDEC 2014). For each land cover covariate, we calculated the proportion within a 100 m buffer. Regardless of study area, all global models also included distance to water (open water bodies and linear water features; Manitoba Remote Sensing Centre 2004 and NLDEC 2014), distance to anthropogenic linear features (roads and trails; National Topographic Data Base 2005, SDSS unpublished data) and terrain ruggedness estimated using package ‘raster’ [41] in R from DEM datasets (Canadian Digital Elevation Data 2006). To represent the declining impact of linear features and water, we log the transformed distance to linear features and distance to water and using the formula ln (distance to *X *+ 1 m).

### (g) Predicting species-specific continuous habitat domain values

Our final step to estimate the relative probability that any given area of the landscape was part of the predator or prey habitat domain was to predict continuous habitat domain values across our study areas. We conducted this prediction from our global bRSF models for each species and season spatially using the predict function in program R.

### (h) Estimating movement synchrony

We used the R package spatsoc [42] to estimate the movement synchrony of prey dyads (caribou and elk). First, we calculated the nearest neighbour at each relocation time *t* and assigned each individual a nearest neighbour dyad (hereafter dyads). To account for small variations in fix time, we rounded each fix time to the nearest 15 min interval. A single individual could be the nearest neighbour to multiple individuals at time *t*, but each dyad at time *t* consisted of a unique combination of two individuals. Ultimately, we are only sampling the behaviour of dyads with the populations, thus our inferences are not linked to specific dyads but instead to dyadic behaviour of the population, we are sampling. We considered dyads able to synchronize movement when within 50 m. Animals could potentially synchronize movements at distances greater than 50 m, however, any apparent synchronization could also be noise in the data, thus we excluded dyads that were greater than 50 m apart. We are not aware of any studies that use empirical data to expressly measure the distance at which individual ungulates in a herd interact. Our use of a 50 m cut-off is thus a trade-off between minimizing the distance and maximizing the number of dyads. We do however note that Kasozi & Montgomery [43] found that 50 m was the most common cut-off value used to distinguish a group. We used the dynamic interaction index (DI) [44] to estimate the movement synchrony between dyads at time *t.* Dyad DI is an estimate of the cohesiveness of two movement vectors (α and β) that incorporates both step distance (*d*) and direction (*θ*), along a scale of −1 (completely asynchronous movement) to 1 (synchronous movement [44]):


$$DI=\left(1-\frac{\left|d_{t}^{\alpha }-d_{t}^{\beta }\right|}{d_{t}^{\alpha }+d_{t}^{\beta }}\right)\times cos\left(\theta_{t}^{\alpha }-\theta_{t}^{\beta }\right).$$

This model of DI does not account for spatial autocorrelation and to our knowledge this remains an unresolved issue in the field. Our method of dyad DI from a subsample of the population is imperfect, there are missing relationships between collared and uncollared animals in the population and among uncollared animals in the population and this could be problematic [45]. We, however, assumed our sample of collared animals in the population was representative of the whole population and we had a wide range of dyad DI values (minimum = −0.96, maximum = 0.99), thus missing social information was less likely to bias our results. It is also worth noting that our dyad DI values were centralized more towards synchrony (Q1 = 0.05 and Q3 = 0.86), thus asynchrony (DI < 0.00) was less represented in our data; however, this is perhaps not surprising given our cut-off value of 50 m, most asynchronous dyads would be filtered out of our dataset within a few steps.

We then used generalized linear regression models to determine the relationship between dyad DI and the predator and prey habitat domains (as predicted from our bRSF models) within our two study areas and seasons. To represent the prey and predator habitat domains of the dyad at time *t* we found the average domain value between individuals in the dyad at time *t*. We ran two models, dyad DI = avg. prey domain + avg. predator domain with and without the interaction term (avg. prey domain × avg. predator domain). Unique dyads were not equally represented in the dataset, which may lead to bias owing to autocorrelation. Thus, we ran models with dyad ID as a random effect using a Bayesian framework with the rstanarm package [46] in R. We compared the performance between the additive and interaction models using leave-one-out cross-validation with the loo package [47,48] in R. We report median parameter estimates, 89% credible intervals, and probablity of direction, calculated using the bayestestR package [49]. To visualize relationships, we plotted the expected values from the posterior distribution using the packages modelr [50], ggplot2 [51], and tidybayes [52] in R.

When we detected an important interaction (probability of direction > 90%) we used the Johnson-Neyman technique [53–55] controlling for false positive rate following Esarey & Sumner [56], using the interactions and visreg packages in R to visualize this relationship [47,57,58]. Note for the visualization of the interactions between prey and predator domains we used a frequentist model without the random effect of dyad ID because the above methods for this assessment were not configured for Bayesian models.

## 3. Results

### (a) Habitat domains and movement synchrony

**Figure 1.**
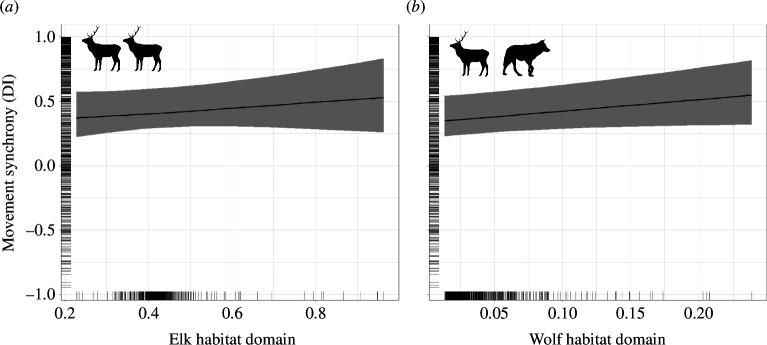
Relationship between elk movement synchrony (DI between two individuals within 50 m) and the elk habitat domain (*a*) and the wolf habitat domain (*b*) during the winter in RMNP, Manitoba, Canada. In the winter, elk movement synchrony was higher in the wolf habitat domain (93% probability of direction), but we did not detect a response in the elk habitat domain. We also did not find support for an interaction of the elk and wolf habitat domains and elk movement synchrony in the winter. Line indicates the expected value of the posterior predictive distribution and band indicates the 89% credible interval. Rug plots along the axes display the distribution of observed values.

**Figure 2.**
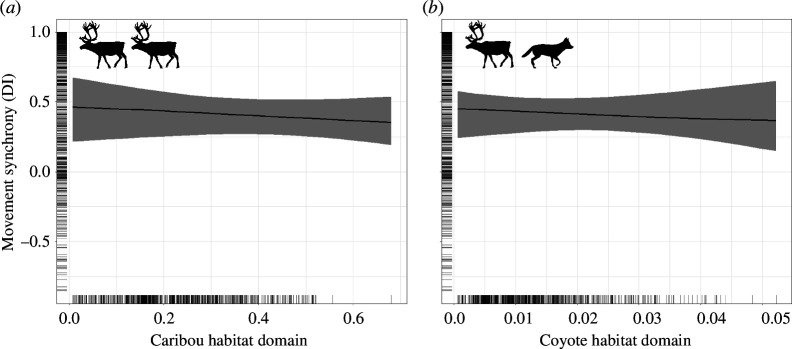
Relationship between caribou movement synchrony (DI between two individuals within 50 m) and caribou and coyote habitat domain during the winter in MRWR, Newfoundland, Canada. In the winter, caribou movement synchrony did not respond to the caribou habitat domain (*a*) but became less synchronous in the coyote habitat domain (*b*; >99% probability of direction). We also detected an important relationship between the interaction of the caribou and coyote habitat domains and caribou movement synchrony in the winter (>99% probability of direction; figure 3). Line indicates the expected value of the posterior predictive distribution and band indicates the 89% credible interval. Rug plots along the axes display the distribution of observed values.

**Figure 3.**
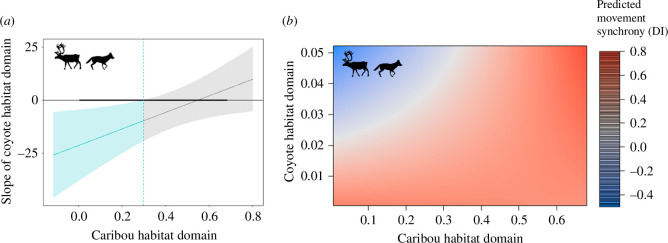
Relationship between caribou movement synchrony ([DI] between two individuals within 50 m) and the interactions between caribou and coyote habitat domains during the winter in Middle Ridge Wildlife Reserve, Newfoundland, Canada. The Johnson-Neyman graph (a) shows the range of caribou habitat domain values at which there was a significant difference in the relationship between coyote habitat domain and caribou synchrony (slope of coyote habitat domain) (area bounded in blue) over the observed caribou habitat domains (thick black line). In the winter, caribou movement synchrony was highest in areas with both high caribou and coyote habitat domains and lowest in areas with high coyote habitat domain but low caribou habitat domain (b). Note these figures were generated from a frequentist model without Dyad ID as a random effect.

Prey movement synchrony during the winter responded to predator habitat domains in both the elk–wolf and the caribou–coyote–bear systems, yet these two systems demonstrated the opposite responses. Elk movement synchrony increased as the wolf habitat domain increased (table 1 and figure 1*b*) whereas caribou movement synchrony decreased as the coyote habitat domain increased but also had a significant interaction between caribou and coyote habitat domains (figures 2 and 3, table 1). Interestingly, in both systems we found that prey movement synchrony did not respond to prey habitat domains during the winter (table 1, figures 1 and 2). Furthermore, elk movement synchrony did not respond to the overlap of elk and wolf habitat domains during the winter (no support for the interaction term). Conversely, caribou movement synchrony did respond to the overlap of caribou and coyote habitat domains during the winter (table 1 and figure 3). We found that the decrease in caribou movement synchrony due to increases in the coyote habitat domain was stronger where caribou habitat domain was lower (β = −17.02 [SE = 5.96] at x̄ – 1 SD caribou habitat domain vs β = −7.50 [SE = 3.92] at x̄ + 1 SD caribou habitat domain; Johnson-Neyman interval at *p* < 0.05 at caribou habitat domain values from 0.01–0.35; note these values were estimated without the random effect of Dyad ID). In other words, we found that the highest predicted caribou movement synchrony during the winter occurred in areas with high probability of being both caribou and coyote habitat domains and the lowest predicted movement synchrony occurred in areas with a high probability of being coyote habitat domain and a low probability of being caribou habitat domain (figure 3*b*; note this figure was generated without the random effect of Dyad ID).

**Table 1.** Median model coefficients (89% credible interval and probability of direction) for models estimating the relationship between prey (elk and caribou) movement synchrony (DI between two individuals within 50 m) and the prey (elk and caribou) and predator (wolf, coyote and black bear) habitat domains in two different study areas, RMNP, Manitoba and MRWR, Newfoundland, Canada. We estimated habitat domains using behaviour-specific resource selection models. For each study area and season (winter and spring), we ran two models of DI and prey and predator habitat domains, one with and one without an interaction term between the predator and prey habitat domains. When the interaction term was supported, we reported the results of that model, otherwise we reported the results of the model without an interaction term.

study area	prey species	season	intercept	model coefficients		
				elk habitat domain	wolf habitat domain	
RMNP	elk	winter	0.23 (0.02 - 0.43)	0.20 (-0.22 - 0.65; 78%)	0.84 (-0.05 - 1.80; 93%)	
				caribou habitat domain	coyote habitat domain	interaction between caribou and coyote habitat domain
MRWR	caribou	winter	0.61 (0.51 - 0.70)	−0.35 (-0.84 - 0.14; 87%)	−21.44 (-33.88 - -8.80; >99%)	45.02 (21.67 - 68.62; >99%)
				caribou habitat domain	coyote habitat domain	black bear habitat domain
MRWR	caribou	spring	0.44 (0.35 - 0.54)	−0.31 (-0.53 - -0.08; 98%)	6.19 (2.58 - 9.93; >99%)	−2.98 (-7.39 - 1.20; 87%)

### (b) Seasonality of prey social response

In our systems, neonate ungulates are substantially more vulnerable to predation during the spring than during the winter. Higher neonate vulnerability during the spring appears to have decreased the gregariousness of the animals we monitored. For example, elk were more likely to have the potential to be social (distance ≤50 m) in the winter (proportion of dyad-times 0.10; 1000 of 10 171) than in the spring (proportion of dyad-times <0.01; 6 of 6568; *Χ* ^2^ = 244.5, *p *< 0.01). We saw a similar but less extreme difference among caribou. Caribou were more likely to have the potential to be social (distance ≤50 m) in the winter (proportion of dyad-times 0.02; 817 of 43 292) than in the spring (proportion of dyad-times 0.01; 665 of 77 812; *Χ* ^2^ = 668.6, *p *< 0.01).

**Figure 4.**
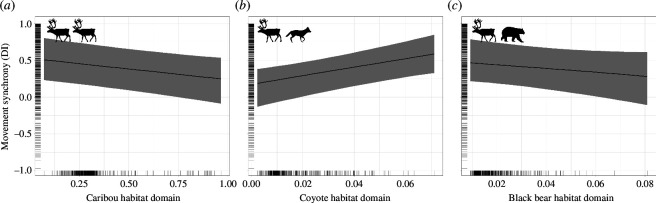
Relationship between caribou movement synchrony (DI between two individuals within 50 m) and caribou, coyote and black bear habitat domains during the spring in the MRWR, Newfoundland, Canada. In the spring, caribou movement synchrony became less synchronous in the caribou habitat domain (*a* ; 98% probability of direction) but became more synchronous in the coyote habitat domain (*b* ; >99% probability of direction) and did not respond to the black bear habitat domain (*c*). We did not find support for a relationship between the interaction of the caribou and coyote habitat domains or the caribou and black bear habitat domains and caribou movement synchrony in the spring. Line indicates the expected value of the posterior predictive distribution and band indicates the 89% credible interval. Rug plots along the axes display the distribution of observed values.

This seasonal difference in vulnerability influenced the relationship between predator habitat domains and prey movement synchrony. For example, caribou decreased movement synchrony within the coyote habitat domain during the winter (see §3.a), yet during the spring caribou movement synchrony increased as coyote habitat domain increased (table 1 and figure 4). Furthermore, while we found evidence of an interaction between the caribou and coyote habitat domains during the winter, we did not find evidence of an interaction between these two domains in the spring. Interestingly, caribou movement synchrony decreased in response to the caribou habitat domain during the spring but not in winter (table 1, figures 2 and 4). Unfortunately, we cannot estimate how elk movement synchrony responded to habitat domains during the spring due to the small sample size of elk dyad-times (*n* = 6) that were within 50 m (our cut-off for the detection of movement synchrony).

### (c) Predator hunting mode and prey movement synchrony

In Newfoundland during the spring, caribou are exposed to two predators with distinct hunting modes (black bear and coyote). While caribou movement synchrony respond to the coyote habitat domain during the spring (see §3.b), there was no response in black bear habitat domain (table 1) and this lack of response was not impacted by an interaction with the caribou habitat domain (no support for the interaction term).

## 4. Discussion

We linked movement synchrony among dyads and predator and prey habitat domains to test the social response of prey to predation risk. Sociality presents a trade-off where groups are more easily detected by predators than solitary individuals [59], but the per capita risk to individuals is lower. Variation in movement synchrony within a group reduces a predator’s ability to target a single individual. The predator swamping or confusion strategies have been used to reduce risk for many species including schooling fish and hatchling turtles [60,61]. Overall, both elk and caribou demonstrated social agreement through cohesive movement within dyads. We expected prey to increase movement synchrony in predator hunting domains. Elk movement was more synchronous within wolf domain in the winter, and caribou were more synchronous within coyote domain in the spring. We expected movement synchrony to be greatest in predator–prey domain overlap which we observed in winter for caribou responding to overlap in foraging habitat and coyote hunting domain. Overall, ungulates increased synchrony in response to cursorial predators and not ambush predators, with some variation between season and system.

Group formation is necessary for movement synchrony to occur, and movement synchrony maintains groups. Prey must first decide whether to form groups at all. Social dyads were rarer in spring than winter for both prey species. In the spring, elk movement was at the extreme of asynchrony as exemplified by the lack of social dyads during this time. The few social dyad observations in the spring probably represent the lower sociality elk expressed during that season. Indeed, the mean annual group size for elk in RMNP is 2–5, and groups tend to be smaller in the spring and summer [62], thus there are fewer opportunities to synchronize movement with conspecifics. Similarly, caribou are also less social in the spring and summer [63,64]. Caribou appear to display two distinct groupings during spring calving: individuals either aggregate in groups on calving grounds or disaggregate off calving grounds, an apparent evolutionary stable state where the probability of calf mortality for each strategy is approximately equal [65]. Therefore, the rarity in spring dyads echoes the findings of previous studies in the same systems, where social groups were larger in winter than spring or summer [62,63].

Ungulates trade-off forage for safety from predators: the resource-risk trade-off is pervasive across scales of responses from the decision to migrate, to within-range selection and patch use, to raising their head to be vigilant [66]. Both species were more social, by forming more dyads, in the winter when resources were less abundant and more patchily distributed than in spring [63,64]. Social antipredator behaviours come with a cost of resource competition and in other systems, elk behavioural responses exemplify this trade-off between predation risk and resource competition in other systems [67,68]. In our study, during the winter elk moved more synchronously in areas where wolves are hunting but did not respond to elk foraging areas. However, in the spring, elk did not form enough dyads to test movement synchrony responses. Elk in RMNP are at low population densities and group sizes are smaller than historically observed. Following the Allee hypothesis, groups in RMNP may not be large enough to reach the threshold of effectiveness of the ‘many eyes’ strategy in the spring. In the winter, caribou movement synchrony increased with predator–prey domain overlap in the winter. When resources are more abundant in spring, caribou moved more synchronously in the coyote habitat domain but did not respond by increasing synchrony in their foraging domain. Caribou on Fogo Island, Newfoundland, travel together with many conspecifics but they forage only with familiar conspecifics [69]. Thus, caribou could be only synchronizing their movements in foraging areas when their dyad partner is familiar, a factor we did not include in our analysis. The benefits of social behaviours seem to differ between movement states, e.g. foraging versus travelling. Vigilance with familiar neighbours and reliable information could matter more when feeding than when moving when detection can occur simultaneously. While travelling, caribou cohesion is influenced most by habitat complexity and not risk [70]. Collective movement is a process of forming and maintaining groups shaped by resource-risk trade-offs and is an emerging path for quantitative empirical tests at fine scales [71].

At a coarser spatial scale, prey may select for vacant domains or spend less time in predator domains when possible [2,34]. Indeed, the areas that prey in our study were foraging and travelling through may be used because of their lower predation risk compared with other areas across the landscape where we did not observe locations (note the absence of data in high predator domain values >0.6 in figures 1*b*, 2*b* and 3*b*). In our study, predator–prey habitat domains were moderately, rather than strongly selected, by both predators and prey. In more tightly linked predator and prey populations, the spatial responses are quite evident. Cougars (*Puma concolor*) have an advantage in landscapes where the same habitat type is resource-rich for both predators and their vicuna (*Vicugna vicugna*) prey, whereas prey have an advantage in more heterogeneous landscapes [3]. The cougar–vicuna system differs from our study systems in that cougar are dietary specialists and require dense vegetation for successful ambushes. When prey cannot evade predators due to high domain overlap the movement response to risk may be more distinct.

Consumptive interaction strengths between predator and prey provide the necessary context for the observed behavioural responses in our study systems. Predation risk for elk is probably greater than for caribou in the winter. Indeed, most elk killed by wolves in RMNP were adults and not calves. For caribou, their vulnerability to predation by coyotes in the winter is lower compared with the spring. Neonate prey are vulnerable to predation, particularly as their mobility is limited during early life [28]. Elk dyads were rare in spring, suggesting the adult female response to the increased vulnerability was to reduce detection rates by decreasing sociality. Caribou remained in dyads during the spring, but increased movement synchrony within coyote predator domains (and did not respond to black bear domains). Group vigilance is beneficial for prey responding to both active cursorial tactics: early detection of predators allows for antipredator responses to be implemented by prey [72]. Furthermore, if prey cannot evade a chase the best alternative is to use their conspecifics to dilute individual risk as they flee from their predator.

We expected the strategy to dilute risk once encountered was mediated by the hunting mode of the predator. Specifically, we expected prey movement synchrony would be more common in habitat domains of coursing predators relative to ambush predators. In agreement with this, elk were synchronous in response to wolves and caribou were more synchronous in response to coyotes than bears in the spring. In the prior century, caribou in Newfoundland persisted without a canid predator for more than 50 years; wolves were extirpated by the 1930s and coyotes colonized in the 1980s. Thus, caribou social behaviour and space use relative to predation, until relatively recently, was largely reflective of ambush and opportunistic predators (e.g. black bears, lynx and eagles) and naive to cursorial predators (e.g. coyotes and wolves). However, in Newfoundland, black bears target younger calves, presenting a short period of risk when calves were young, while coyotes consume older and larger calves, indicating prolonged risk [26]. Despite this, the differences in movement response were more distinct between predator hunting modes and seasons than between prey species. Furthermore, in RMNP, elk are no longer the primary prey of wolves, but wolves are still spatially selecting for elk catchability when hunting [19]. Both predator and prey have maintained responses that echo the tightly linked predation relationship from the past. Therefore, the strength of consumption interactions shapes the strength of the social movement responses, but prey appear to both conserve their risk responses to cursorial predators whether historical or emergent in the system.

There arise several future avenues for exploration of fine-scale prey social responses to risk:

— *Definition of domain and risk*. We used hunting and foraging-specific habitat selection analyses to estimate predation risk on the landscape; however, an alternative measure of predator–prey domain overlap and predation risk is from direct encounters [73]. Estimating predation risk on the landscape would require more fine-scale movement data [74] or intensive field observations of behaviours including encounters, chases and success.
— *Determine the effect of diet specialization and joint-spatial anchors*. Future work should examine movement synchrony changes within predator–prey domains for specialized predators or in areas where habitat domains explicitly intersect (i.e. joint-spatial anchors [3]). Prey are expected to respond more strongly to predators with narrow domains [23] and predators that are more lethal per encounter [75].
— *Incorporate fine-scale temporal variation*. Evaluating the response of prey movement synchrony to predator domains at a finer temporal scale may highlight a shift in responses. For example, our estimates of predation risk were based on predator space use during the entire season, however, caribou calf predation risk from bears decreases as calves age (and grow) and bears switch to different prey resources [26]. Conversely, predation risk from coyotes, while lower than bear predation risk in the early calving season, is more constant throughout the spring and summer [27]. At a diel temporal scale, elk in Yellowstone National Park have a spatially explicit perception of predation risk that depends on the predator species and the predator activity pattern [34].

Ultimately, our study demonstrates that the exploration of habitat domains can be successfully completed using remotely sensed data and integrated with contemporary techniques in other fields to test ecological mechanisms [76,77].

There is safety in numbers and prey must move together to stay together; accordingly, we used movement ecology as a test of behavioural responses of predation on sociality. Prey must dynamically balance the costs and benefits of sociality by assessing a series of internal and external influences. Both gregarious ungulates increased synchrony in response to risk from cursorial predators when predation pressure was higher. We emphasize the importance of considering not only how spatial variation in predation risk can impact the use of social behaviour as an antipredator strategy by prey but also the role of other factors such as prey vulnerability and predator hunting mode.

## Data Availability

The data and code supporting this manuscript are available on Github and archived in Zenodo [78,79]. Supplementary material is available online [80].
